# Intra-hepatic arterial pseudoaneurysm causing life-threatening upper gastrointestinal bleed after removal of biliary drainage catheter

**DOI:** 10.2349/biij.5.3.e20

**Published:** 2009-07-01

**Authors:** M Taneja, R Lo, MG Sebastian, PKH Chow

**Affiliations:** 1 Interventional Radiology Centre, Singapore General Hospital, Singapore; 2 Department of General Surgery, Singapore General Hospital, Singapore

**Keywords:** Hepatic artery pseudoaneurysms, internal-external biliary drain insertion, upper gastrointestinal bleed, Whipple’s operation, pancreatic cancer

## Abstract

Hepatic artery pseudoaneurysms are an uncommon complication of percutaneous biliary drainage catheter insertion. The authors report a case of a hepatic artery pseudoaneurysm following percutaneous internal-external biliary drain insertion. This led to massive haemobilia when the catheter was removed and presented clinically as life-threatening upper gastrointestinal bleed. The clinical and imaging manifestations are discussed along with the management of the patient.

## INTRODUCTION

There are various causes of massive haematemesis with peptic ulcer disease and variceal haemorrhage being the more common underlying conditions. Haemobilia is a less well-known cause of critical haematemesis [1]. The authors report a case of haemobilia arising from a hepatic artery pseudoaneurysm, which developed as a complication of percutaneous biliary drainage catheter insertion, leading to massive upper gastrointestinal haemorrhage and collapse of the patient when the catheter was removed. This case illustrates a number of important clinical points namely the value of early clinical recognition of the underlying complication, the useful role of CT angiography in detection and management of hepatic artery pseudoaneurysms, the apparent lack of correlation between lesion size and clinical presentation and the role of endovascular treatment in management of these patients.

## CASE REPORT

A 54-year-old, previously healthy, male patient investigated for progressive jaundice was found to have a carcinoma of head of pancreas with dilated proximal bile ducts. The patient developed cholangitis and an 8F internal-external biliary drainage catheter was placed, through the left intra-hepatic bile duct approach.

He subsequently underwent a Whipple’s operation 3 days later. During the operation, the tumor was found to be adherent to the superior mesenteric artery (SMA) with partial venous invasion at the confluence of superior mesenteric vein (SMV) with portal vein (PV). The tumor was dissected off the SMA, sleeve resection of the SMV and PV junction was performed and the defect closed with primary reconstruction. The biliary drainage catheter was left as a stent across the hepatico-jejunal anastomosis.

Postoperative recovery was uneventful. Two weeks post-surgery, he was brought into the angiography suite for cholangiogram through the existing left biliary drainage with a view to catheter removal, prior to discharge from the hospital.

**Figure 1:**
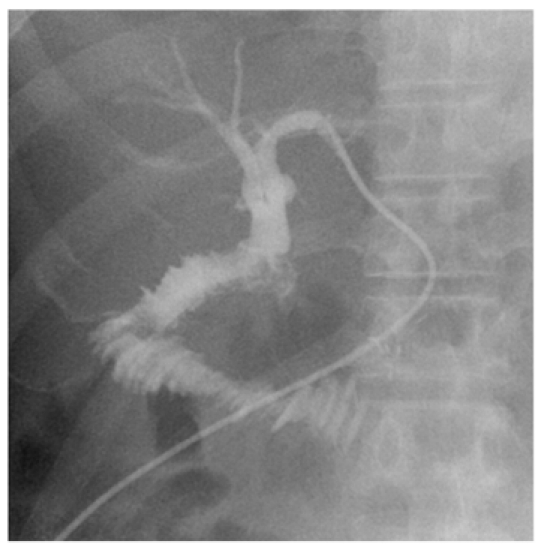
Cholangiogram performed through retained left internal-external biliary drain 2 weeks post-procedure shows patent anastomosis with free drainage of contrast into small bowel loops.

The cholangiogram revealed a patent anastomosis with good drainage into bowel (Figure 1). The biliary drain was removed on the angiographic table upon completion of the cholangiogram. Minimal oozing of blood from the drainage catheter entry site on the skin in the midline was observed, which stopped with compression.

While preparation was being done to transfer the patient from the angiographic table, he became increasingly unwell. Within ten minutes of tube removal, there was an episode of massive haematemesis. He became increasingly hypotensive and tachycardic, and collapsed. Emergency resuscitation and intubation was performed, with the patient urgently evacuated to the emergency operating room. He was stabilised while preparations were being made to operate on him again. One of the major possibility considered for the patient’s presentation was bleeding from the breakdown at the portal venous reconstruction site. While this was being done, emergent endoscopy and ultrasound of abdomen was performed. There was no source of bleeding identified on endoscopy, with no free fluid in the abdomen or pelvis.

As the patient stabilised with blood transfusion and continued to remain stable with active resuscitative measures, massive haemobilia from pseudoaneurysm was considered, especially since there was no free fluid in the abdomen or pelvis on ultrasound. CT angiogram was performed in order to identify the source of bleeding more accurately before more invasive intervention was undertaken.

**Figure 2:**
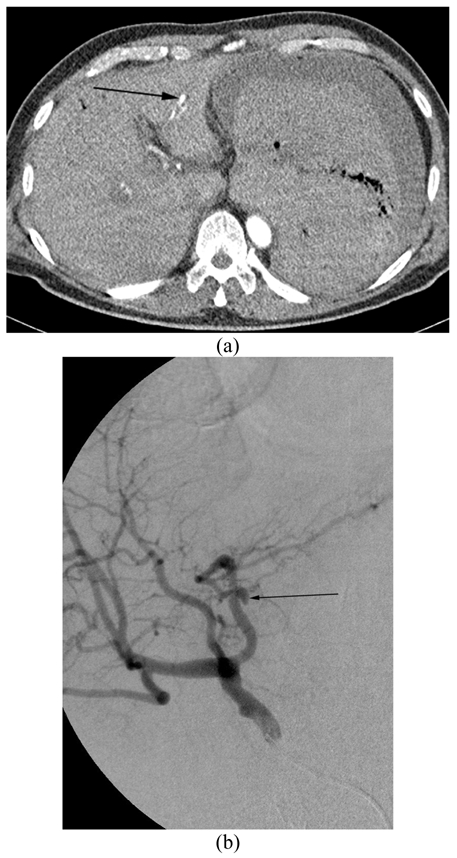
(a) CT angiogram shows 1 cm left hepatic artery pseudoaneurysm (arrow) within left lobe of liver. (b) CT angiogram immediately below the above lesion demonstrates active extravasation of contrast in the adjacent bile duct (arrow).

The arterial phase of CT angiogram revealed a 1 cm pseudoaneurysm arising from one of the intraparenchymal left hepatic artery branches with active contrast extravasation into an adjacent bile duct (Figure 2a and 2b).

**Figure 3:**
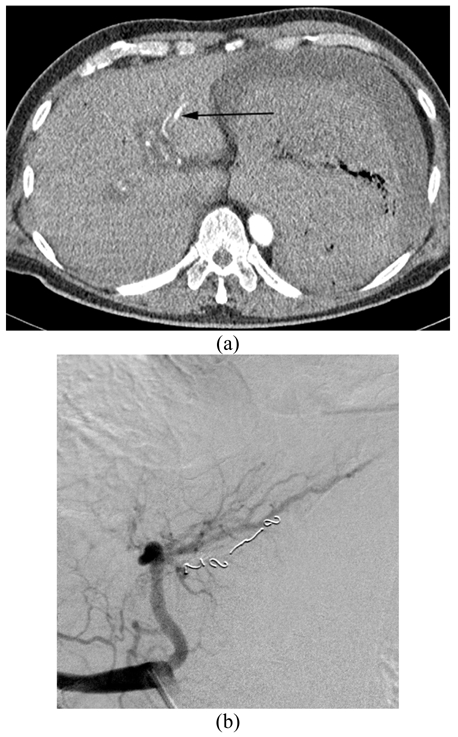
(a) Selective angiogram confirms a small pseudoaneurysm (arrow) arising from segment 3 hepatic artery. (b) Post-embolisation angiogram demonstrates complete exclusion of the pseudoaneurysm.

On the basis of imaging and because of the intrahepatic location of the lesion, a decision was made to manage the lesion through an endovascular approach. Selective catheterisation and embolisation of the lesion was performed with microcoils with complete angiographic exclusion of the pseudoaneurysm (Figure 3a and 3b). The patient recovered well over the next few days and was discharged from the hospital 10 days later.

## DISCUSSION

Biliary drainage catheter insertion is a well-recognised cause of haemobilia related to underlying vascular complication of the procedure [1]. Clinically, suspicious signs of presence of these complications include blood stained bile in the drainage bag, oozing of blood around the catheter entry site on the skin, significant gastrointestinal bleeding and falling haemoglobin levels, and bleeding from the tract at the time of tube change or removal [2].

The acute presentation in this patient with massive haemobilia and haematemesis leading to collapse is quite unusual, especially since the bile draining into the connecting bag prior to removal of the catheter did not suggest prior bleeding. The patient had also recovered well from surgery and there were no risk factors such as deranged clotting profile.

Intrahepatic arterial injury related to biliary drainage procedures have been reported to have an incidence of about 2% [3], with suggestion of the complication being higher in biliary drainage catheters inserted from the left compared to the right hepatic lobe [4]. However, with studies documenting higher patient comfort and acceptability for left hepatic lobe interventions [5], and technically easier direct image-guided approach, left intrahepatic duct is the preferred option for biliary access and drain insertion in distal lesions of the biliary tree with communicating right- and left-sided ducts. Certainly, this is the preferred approach at the authors' institute.

In this patient, an experienced operator placed the left internal-external biliary drain following biliary access into segment 3 bile duct under ultrasound guidance. No difficulty was encountered during the procedure and the drain was left inside the patient for 18 days. The drainage catheter caused injury to the wall of the adjacent left hepatic artery, and tamponaded the site, until its removal led to massive bleeding.

The case emphasises the importance of clinical acuity and the extremely useful role of CT angiography in evaluating these patients. CT angiogram has a well-established role in patients presenting with gastrointestinal haemorrhage not detected by endoscopy, especially when the patient is hemodynamically stable [6, 7]. CT angiogram not only detected the vascular abnormality accurately in this case, but was also pivotal to the decision for endovascular intervention. The intra-hepatic pseudoaneurysm and the active extravasation of contrast into the biliary duct were well identified. Reviewing the thin section images carefully at the time of the scan is important to establish an accurate diagnosis. Also of clinical interest is the apparent discordance between the size of the lesion and the clinical presentation of the patient.

Hepatic pseudoaneurysms can be effectively treated by the endovascular approach [8, 9]. Embolisation is relatively safe and can be performed successfully in the majority of these patients. Surgery has minimal role in such cases except in patients with aberrant or extremely tortuous vessels where the target lesion cannot be accessed.

Various embolisation agents such as coils, N- butyl cyanoacrylate (NBCA), gelfoam and polyvinyl alcohol (PVA) particles are available for treating these lesions. In the authors' experience, coils are highly effective and provide safe, durable embolisation [10]. In embolizing pseudoaneurysms, care should be taken to occlude both the “front and back doors”, to prevent the risk of retrograde recanalisation of the pseudoaneurysm at a later stage. A few accurately placed microcoils are usually sufficient for effectively occluding pseudoaneurysms.

In the post-embolisation period, patients frequently stabilize haemodynamically soon after the procedure. Isolated episodes of further gastrointestinal haemorrhage of altered blood contents are not unusual in the post- procedure period, and should not be of concern as long as the haemodynamic parameters are stable.

In conclusion, biliary drainage catheter related iatrogenic hepatic arterial injury can be life threatening. A high index of suspicion ensures prompt diagnosis and management of these patients.
